# Versatile-in-All-Trades: Multifunctional Boron-Doped Calcium-Deficient Hydroxyapatite Directs Immunomodulation and Regeneration

**DOI:** 10.1021/acsbiomaterials.2c00242

**Published:** 2022-06-16

**Authors:** Ahmet Engin Pazarçeviren, Sema Akbaba, Zafer Evis, Ayşen Tezcaner

**Affiliations:** † Department of Engineering Sciences, Middle East Technical University, Ankara 06800, Turkey; ‡ Department of Biotechnology, Middle East Technical University, Ankara 06800, Turkey; § Center of Excellence in Biomaterials and Tissue Engineering, Ankara 06800, Turkey

**Keywords:** boron, hydroxyapatite/tricalcium phosphate, immunomodulation, angiogenesis, osteogenesis

## Abstract

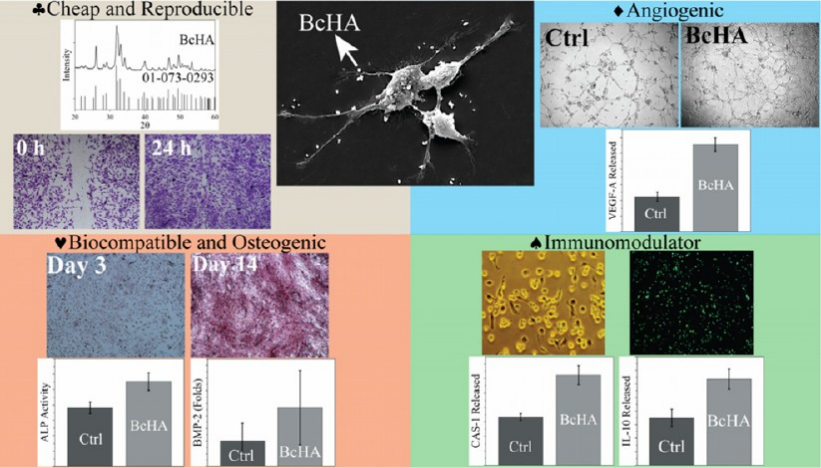

Osseointegration of implants depends on several intertwined factors: osteogenesis, angiogenesis, and immunomodulation. Lately, novel reinforcements allowing faster bonding with osseous tissue have been explored intensively. In this study, we hypothesized the use of boron as a major multifunctional ion to confer versatility to calcium-deficient hydroxyapatite (cHA) synthesized by a wet precipitation/microwave reflux method. By synthesis of boron-doped calcium-deficient hydroxyapatite (BcHA), we expected to obtain an osteoimmunomodulatory and regenerative nanoreinforcement. BcHA was found to possess a pure HA phase, a greater surface area (66.41 m^2^/g, *p* = 0.028), and cumulative concentrations of Ca (207.87 ± 6.90 mg/mL, *p* < 0.001) and B (112.70 ± 11.79 mg/mL, *p* < 0.001) released in comparison to cHA. Osteogenic potential of BcHA was analyzed using human fetal osteoblasts. BcHA resulted in a drastic increase in the ALP activity (1.11 ± 0.11 mmol/gDNA·min, *p* < 0.001), biomineralization rate, and osteogenic gene expressions compared to cHA. BcHA angiogenic potential was investigated using human umbilical cord vein endothelial cells. Significantly, the highest VEGF-A release (1111.14 ± 87.82 in 4 h, *p* = 0.009) and angiogenic gene expressions were obtained for BcHA-treated samples. These samples were also observed to induce a more prominent and highly branched tube network. Finally, inflammatory and inflammasome responses toward BcHA were elucidated using human monocyte-derived macrophages differentiated from THP-1s. BcHA exhibited lower CAS-1 release (50.18 ± 5.52 μg/g_DNA_ μg/gDNA) and higher IL-10 release (126.97 ± 15.05 μg/g_DNA_) than cHA. In addition, BcHA treatment led to increased expression of regenerative genes such as VEGF-A, RANKL, and BMP-2. In vitro results demonstrated that BcHA has tremendous osteogenic, angiogenic, and immunomodulatory potential to be employed as a “versatile-in-all-trades” modality in various bone tissue engineering applications.

## Introduction

Osseointegration of an implant is a unique process arising as a result of successful bone-making processes occurring concurrently with angiogenic and immunomodulatory processes.^1^ Especially for the next-generation bioceramics, multifunctionality in addition to induction of osteogenesis resulting in a lower immunological response and a greater rate of vascularization around the implant have been prioritized.^2^ Because bioceramics produced without additional osteogenic ions had been ineffective in triggering a rapid osteogenic response, various ions have been utilized as dopants to modulate the cross-talk between osteogenic, angiogenic, and immunomodulatory pathways.^3−6^ Moreover, foreign ions incorporated into bioceramics such as calcium-deficient hydroxyapatite (cHA) demonstrated high stability.^7−9^ cHA possesses highly similar microstructural properties having the formula (10 – *x*)Ca·*x*HPO4·(6 – *x*)PO_4_·(2 – *x*)OH and calcium to phosphate ratio (1.3 < Ca/P < 1.67) with natural bone apatite.^10^ Moreover, it was demonstrated that cHA has lower solubility than stoichiometric HA (Ca/P = 1.67); thus it is expected that it can release bioactive ions quite faster.^11^

Owing to complex and hierarchical nature of osseous tissue, bone healing could be enhanced and regeneration may be supported via the use of highly bioactive and multifunctional bioceramics forming a suitable microenvironment. Lately, B appeared to be an important dopant in the form of nanotubes,^12^ in bioglasses,^13,14^ and in bioceramics^15,16^ to improve osteogenic potential. In a recent study, B was shown to induce transcription factor 7-like 2 (TCF7L2) in downstream of the β-catenin/Wnt signaling pathway to promote an increased osteogenic response of MC3T3-E1 preosteoblasts.^17^ In addition, several other studies revealed that B could prompt the angiogenic response of ST2 cells derived from mouse bone marrow.^18,19^ B uptake through sodium boron cotransporter 1 (NaBc1) was proposed to stimulate human umbilical cord vein endothelial cells (HUVECs) and reinforce angiogenesis through soluble vascular endothelial growth factor receptor (VEGFR) colocalization.^20^ However, the innate immunological response with respect to inflammasome production and inflammatory reaction, another important key factor in bone regeneration, has been overlooked. In the literature, there are only a few studies that analyzed the inflammatory response toward B-doped bioceramics.^21,22^ Although the inflammasome production mechanism in addition to inflammation has been studied meticulously for the elucidation of the immunological response to various biomaterials, the innate immune reaction toward bioceramics has not been fully appreciated.

**Figure 1:**
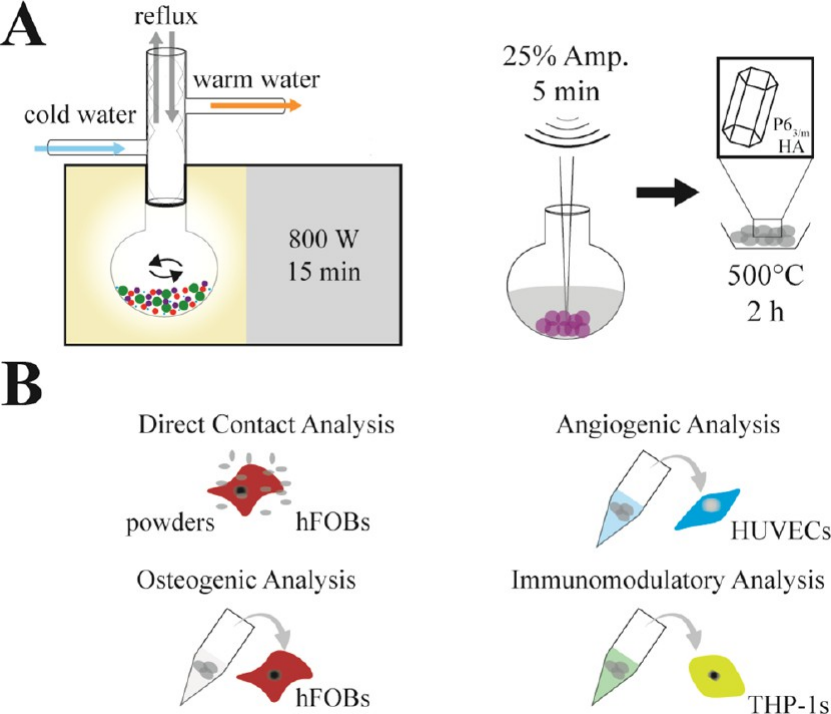
Methods followed in this study. Wet precipitation/microwave reflux was employed to produce cHA and BcHA which were then downsized using a probe sonicator (A). Biological properties of samples were characterized in terms of extracts obtained in different media (B).

We hypothesized that B has the ability to direct responses toward cHA in terms of three interconnected yet separate biological phenomena (Figure 1). Boron-doped calcium-deficient hydroxyapatite (BcHA) could prompt good bone-specific enzyme activity, osteogenesis, and angiogenesis and may alleviate the innate immune response by promoting a balance in proinflammatory and prohealing pathways. cHA and BcHA conditioned media were employed to study their effects on the biological responses. Effects of B on various effectors and genes were studied to characterize the effect of boron on a moderately crystalline cHA structure using human fetal osteoblasts (hFOBs), HUVECs, and human monocyte-like cells (THP-1s) for the first time. Human fetal osteoblasts have been used as a model osteoblastogenic cell line in the literature for many decades.^23,24^ Moreover, studies involving an angiogenic marker and tube formation analyses have been using HUVECs as an endothelial model cell line.^25,26^ Lately, human monocyte cell line, THP-1-ASC-GFPs or simply THP-1s, which can stably express apoptosis-associated speck-like protein containing a caspase activation and recruitment domain (ASC) conjugated with a green fluorescent protein (GFP), has been widely used to monitor inflammasome production.^27^ THP-1s were also used in macrophage (MΦ) response analysis toward proinflammatory (M1) and prohealing (M2) directions.^28^

In our previous study, Pazarçeviren et al. demonstrated that the B dopant provided a significant improvement in microstructural and biological properties of a biphasic calcium phosphate species (HA/β-TCP) with 5% molar B dopant in comparison to 1, 2, 3, and 10 molar% B-doped counterparts.^29^ In this study, we produced B-doped cHA having an initial Ca/P ratio of 1.6 to further characterize the effect of B on the biological properties of bone-similar synthetic moderately crystalline HA in vitro. To determine the osteogenic properties, the activity of ALP, an osteogenic enzyme, was quantified, and accompanied changes in osteogenic gene expressions were also studied using hFOBs. Tube formation, VEGF-A production, and angiogenic gene expressions were quantified using HUVECs. Finally, inflammatory and inflammasome responses in terms of gene expressions and their translation into respective proteins were determined to elucidate the effect of B at the junction of critical pathways acting in osteoimmunomodulation using THP-1s.

## Materials and Methods

Calcium nitrate dihydrate (Ca(NO_3_)_2_.2H_2_O), diammonium phosphate ((NH_4_)_2_HPO_4_), boric acid (H_3_BO_3_), ammonia (NH_4_OH), acetic acid (glacial), l-ascorbic acid, β-glycerophosphate, and dexamethasone were purchased from Sigma, USA. DMEM:F12, fetal bovine serum, penicillin–streptomycin cocktail, RPMI 1640, EndoGo XF, and EndoGoXF supplements were purchased from Biological Industries, Israel. PMA and Normocin were obtained from Invivogen, USA. Other chemicals and solutions used in the study are reagent-grade.

### Synthesis of cHA and BcHA

Calcium-deficient Hap (cHA) and 5% molar B-doped cHAP (BcHA) were synthesized through the microwave reflux method.^30^ Calcium nitrate dihydrate (Ca(NO_3_)·2H_2_O) and diammonium phosphate ((NH_4_)_2_HPO_4_) were used as Ca and P sources, respectively. Boric acid (H_3_BO_3_) was utilized as the B source. The Ca source (615 mM) was dissolved in deionized water, and the initial pH was set to 10 by addition of ammonia (NH_4_OH). For the production of cHA, the Ca source was added dropwise to the solution of the P source (385 mM) prepared in dH_2_O. For the production of BcHA, a mixture of P (335 mM) and B (50 mM) sources was added dropwise while pH was stably held between pH 9 and 10. After a white colored suspension formed, the mixture was aged in a microwave oven for 15 min under 800 W in the reflux system. The wet cake was then centrifuged at 8000*g* for 3 min at 4 °C, suspended in pure acetone, and homogeneously downsized using a probe sonicator at 25% amplitude for 2 min. After that, powders were dried at 100 °C and calcined at 500 °C for 2 h.

### Microstructural Characterization

X-ray diffraction (XRD) spectra of cHA and BcHA were obtained between 2θ at 20–60° with 0.02°/s under 40 kV and 30 mA CuKα radiation (XRD, PANalytical Empyrean, USA). Crystallinity (*X*_c_), lattice parameters, volume, and density of cHA and BcHA were calculated using supplementary eqs (S1–S4) given in the Supporting Information. Given equations were employed for hexagonal *P*63/*m* conformation as determined by XRD analysis. Functional groups were determined using ATR/FTIR in the midrange of 4000–400 cm^–1^ at 2 cm^–1^ resolution with an average of four scans (Bruker IFS66/S, USA). Powders in 1 mg/mL were homogeneously mixed in pure ethanol, dripped on a carbon tape, and coated with gold. Morphology of cHA and BcHA was observed by scanning electron microscopy (SEM) at high vacuum (FEI Quanta 600, Thermo Fisher, USA).

### Composition, Ion Release Profiles, and Surface Area of cHA and BcHA

The particle size was determined with 100 mg powder dispersed in 1 mL (*n* = 3, Malvern Mastersizer 2000, UK). The elemental composition of cHA and BcHA powders was detected using ICP (ICP-MS, Perkin Elmer Optima 4300DV, USA) after completely dissolved in 10% (v/v) nitric acid. Prior to further analyses, powders were heat-sterilized at 200 °C for 2 h, and amounts of B, Ca, and P released from samples (2 g/10 mL) in deionized water (dH_2_O) supplemented with 0.2% (w/v) sodium azide (NaN_3_) was determined by ICP (*n* = 3). This medium was selected as the release medium to decrease the matrix effect occurring due to high metallic ion concentration, which usually prevents the quantification of total B released.^31^ Total ion release was given cumulatively for each sample at time periods and normalized to the initial dry weight of the given sample powder. The specific surface area (SSA, m^2^/g) of particles was determined by multipoint Brunauer–Emmett–Teller (BET) analysis (*n* = 3, Autosorb-6, Anton Paar, Austria). Before analysis, particles were degassed at 150 °C for 16 h.

### Cell Culture Studies

For in vitro cell culture analyses, conditioned media having cHA and BcHA release products were used. cHA and BcHA were sterilized at 200 °C for 2 h and added to various cell culture media of different compositions at a concentration of 0.1 g/mL (Table S1), and extracts were collected after 24 h incubation. Moreover, cell types used in the analyses are also given in Table S1. Except for THP-1, all cells were cultured until 80–90% confluency and trypsinized for use. THP-1s seeded at a concentration of 2 × 10^6^ cells/mL concentration were cultured in flasks until the desired number of cells for immunomodulation study was reached (64 M cells in 32 mL media).

### Cell Proliferation and Migration Studies

#### Cell Proliferation Assay

hFOB cells (p. 8, 5 × 10^3^/well in 48-well plates) were incubated with extracts obtained in growth media for 1, 4, and 7 days (*n* = 8). Cell viability was measured at the end of each incubation period using Alamar Blue viability assay (Thermo Fisher, USA). Briefly, media were discarded, cells were rinsed with phosphate buffered saline (PBS, 0.01 M, pH 7.4), and Alamar Blue viability agent prepared in growth medium (10% v/v) was added into the wells. Cells were incubated for 2 h at 37 °C, 95% CO_2_ in an incubator (Panasonic Incusafe, Japan). Afterward, aliquots were collected, and optical densities (ODs) at 570 nm and 600 nm were measured. hFOB proliferation was calculated using the formula provided by the supplier.

#### Osteoblast Migration Assay

Cells seeded in six-well plates at a density of 1 × 10^6^ cells/well were incubated until reaching 95–100% confluency. After that, hFOBs were scratched in a single straight line using a 20 μL pipette tip and rinsed twice with PBS (0.01 M, pH 7.4) to remove debris and detached cells.^32^ hFOBs were then incubated in conditioned media for 8 and 24 h to observe the effect of cHA and BcHA on hFOB migration (*n* = 3). Growth media-added hFOBs were used as the control. After 24 h, hFOBs were fixed and stained with 0.5% w/v crystal violet in 1:4 v/v methanol in water for 30 min. Purple-stained cells were visualized using a phase contrast microscope at 4×, and initial and final scratch widths at two different points for each scratch sample were measured.

#### Cell Morphology after Direct Contact

Employing similar conditions to those above, hFOBs were made to interact directly with 100 μg/mL powder for 1 and 7 days after seeding on glass coverslips having 1 cm diameter (*n* = 3). After incubation, media were discarded, wells were vigorously rinsed with PBS twice to remove unattached particles, and cells were fixed and stained with crystal violet prepared in 0.5 wt% at 1:4 (v/v) methanol in dH_2_O for 30 min. Then, hFOBs were visualized using a phase contrast microscope (Eclipse TS100, Nikon, Japan). Additionally, the presence of particles in and around of hFOBs was studied. After exposure to another batch of cHA and BcHA, hFOBs were fixed with 2.5% (v/v) glutaraldehyde in dH_2_O for 4 h, freeze-dried at −80 °C for 4 h, coated with Au for 4 min, and visualized by SEM (FEI Quanta650, USA) equipped with a backscatter detector.

### Osteogenesis Study

#### ALP Activity and Quantitative Real-Time Polymerase Chain Reaction (qPCR) Assays

hFOBs were seeded in 2 × 10^5^ cells/well in 24-well plates. After incubation for 24 h, cHA and BcHA extracts in osteogenic media were added, and cells were cultivated in these conditioned media for 2 weeks (*n* = 6). Every 2 days, media change was done with the extracts. At the end of each week, sample media were discarded and rinsed with calcium- and magnesium-free PBS (0.01 M, pH 7.4). Then, lysis buffer composed of 0.1% Triton X-100 in 0.2 M carbonate buffer was added on cells, and plates were awaited at −80 °C. After two freeze–thaw cycles (−80 to 25 °C), aliquots were collected aseptically and incubated with the substrate working solution (10 vol para-nitrophenyl phosphate (pNpp), 20 vol dH_2_O, and 1 vol MgCl_2_·6H_2_O in dH_2_O) for 1 h at 37 °C, and absorbance of the solutions was measured at 405 nm (OD_405_). Total ALP concentration of the lysates was determined using the calibration curve constructed with different concentrations of *p*-nitrophenol. In addition, total DNA of the lysates was determined using 300 times diluted Picogreen DNA dye (Thermo Fisher, USA) prepared in Tris-EDTA buffer (TE, pH 8.5). Dye was added to the aliquots in 1:1 v/v ratio; after 5 min of incubation, fluorescence intensities (Excitation: 488 nm and Emission: 538 nm) were measured, and DNA amounts were determined in accordance with the supplier’s protocol (Thermo Fisher, USA). At the end of analysis, specific ALP activity (mmol/g_DNA_.min) was calculated by normalizing ALP concentration obtained at the end of 1 h incubation with pNpp to the total DNA content of each sample. Furthermore, total RNA was isolated in accordance with the supplier’s protocol (*n* = 4, High Pure mRNA Isolation Kit, Roche, Switzerland) for qPCR analysis of ALP, OSX, OCN, COL1A1, BMP-2, RUNX2, and RANKL. Isolated RNA quality was measured and quantified, and cDNAs were synthesized (Applied Biosystems PCR System 9700, USA) in accordance with the supplier’s protocol (Transcriptor High Fidelity cDNA Kit, Roche, Switzerland). Using an SYBR Green RT-PCR Kit, cDNAs were combined with the primers given in Table S2, and qPCR was conducted (Roche Lightcycler 480, Switzerland). Amplification reactions were performed for 45 cycles, and the cycle number at detection threshold (*C*_t_) for each sample was determined. β-actin was used as the housekeeping gene to determine cycle threshold (Δ*C*_t_) values for each sample. Relative changes in gene expression of samples were calculated by the 2^–ΔΔ*Ct*^ method.

#### Biomineralization Analysis

Cells seeded on 12-well plates at a density of 4 × 10^5^ cells/well were incubated in the conditioned media over 2 weeks’ period. At the end of each week, media were discarded, and samples were rinsed with PBS and incubated with 2% w/v Alizarin Red (Sigma, USA) at pH 4.2 for 30 min at room temperature. Then, the dye was removed, and cells were rinsed with dH_2_O thrice. Purple-red calcium deposits around the cells were visualized using a phase contrast microscope at 4× and photographed with the conventional camera. Alizarin Red staining (ARS) was quantitated using the colorimetric method.^33^ Briefly, 10% v/v acetic acid was added to the stained wells, heated up to 85 °C to solubilize ARS, and then treated with 10% v/v NH_4_OH to develop the characteristic pinkish color. Aliquots were taken, and their OD_405_ was measured. With known concentrations of calcium chloride, a calibration curve for ARS quantitation was constructed, and calcium amounts deposited by the cells were determined and normalized to DNA content.

### Angiogenesis Study

#### Tube Formation Assay

Growth factor reduced and LDEV-free Matrigel basement membrane matrix (Corning, USA) was thawed at +4 °C, placed in 96-well plates (50 μL/well), and polymerized in an incubator for 30 min prior to cell seeding. HUVECs were seeded in Matrigel-coated wells at a density of 40.000 cell/150 μL, and subsequently cHA and BcHA conditioned angiogenic media (conditioned for 24 h) were added to the wells (*n* = 3). At the end of 4 h, HUVECs were observed using a phase contrast microscope and photographed at two separate locations in each sample well (*n* = 6). The number of master junctions, meshes, mesh sizes, mesh areas, and master segment lengths of the images taken at the center of wells were analyzed using ImageJ (NIH, USA) with Angiogenesis Analyzer add-on.^34^

#### VEGF-A Release and qPCR Analysis

HUVECs were incubated in cHA and BcHA conditioned media for 4 h (*n* = 4). Media were then collected and stored at 4 °C while cells were lysed using ice-cold PBS (0.01 M, pH 7.4) and subjected to subsequent freeze–thaw cycles. Lysates and supernatants were combined in 1:1 v/v ratio and total VEGF-A production was quantified using a human VEGF-A ELISA kit (CSB-E11718h, Cusabio, China). Concurrently, cellular viability was also measured using MTT assay (*n* = 8, Sigma, USA). Supplier’s protocol was followed and HUVECs seeded in tissue culture plates served as the positive control in both VEGF-A detection and MTT assays. Positive-control cell viability was assumed 100%. Additionally, angiogenic VEGF-A, VEGFR2, and eNOS gene expressions of cells treated with the conditioned media (*n* = 4) for the same period of time were determined. Primers used in the qPCR study are given in Table S2.

### Immunomodulation Study

#### Immunomodulatory Protein Release and qPCR Analysis

THP-1 cells expressing GFP-conjugated ASCs were incubated in the monocyte growth medium. The cells were grown in monocyte medium until reaching the desired cell number. Prior to addition of cHA and BcHA conditioned media, THP-1 cells were seeded in 24-well plates at a density of 0.5 million/well and incubated for 24 h. Then, PMA was added (10 nM), and cells were differentiated into monocyte-derived macrophage-like cells and became adherent. After another 24 h of incubation, cells were incubated in the conditioned media for 24 h. Then, aliquots from each sample (*n* = 4) were collected, centrifuged at 8000*g* for 10 min, and assayed with a human CAS-1 ELISA kit (E4588, Biovision, USA). At the same time, aliquots were tested using a human IL-10 ELISA kit (CSB-E04593h, Cusabio, China). Afterward, media were discarded, cells were rinsed with PBS twice, and MTT reagent (*n* = 4) was added. Relative cell viability was calculated for each group in accordance with the supplier’s protocol (Sigma, USA). Additionally, total RNA of another set of cells cultured under the same conditions was isolated as explained previously. Gene expression of CAS-1, NfKB, IL-1β, IL-10, iNOS, RANKL, BMP-2, and VEGF-A was analyzed (*n* = 3). Primer sequences are given in Table S2.

#### Morphological Analysis

During immunomodulation analysis, the innate immune response to cHA and BcHA was qualitatively analyzed. Morphology of PMA-treated THP-1s in the conditioned media was examined under the phase contrast microscope (10× magnification), and ASCs generated during incubation periods were visualized and semiquantitatively measured under a fluorescence microscope (10× magnification, Zeiss Axio Scope, Germany) at 488 nm excitation/509 nm emission for GFP. For all analyses, Ctrl are THP-1s that have not been incubated with conditioned media.

### Statistical Analysis

Data were presented in mean ± standard deviation. All data were tested by the Shapiro–Wilk normality test and analyzed for homogeneity of variances using Levene’s test prior to multiple comparison analysis. Statistical differences among groups were determined by one-way analysis of variance employing Tukey’s post hoc test (*p < 0.05, **p < 0.01, and ***p < 0.001). Results of all qPCR results and ELISA assay data were analyzed in pairs by an independent pairwise two-tailed t-test assuming equal variances.

## Results

**Figure 2:**
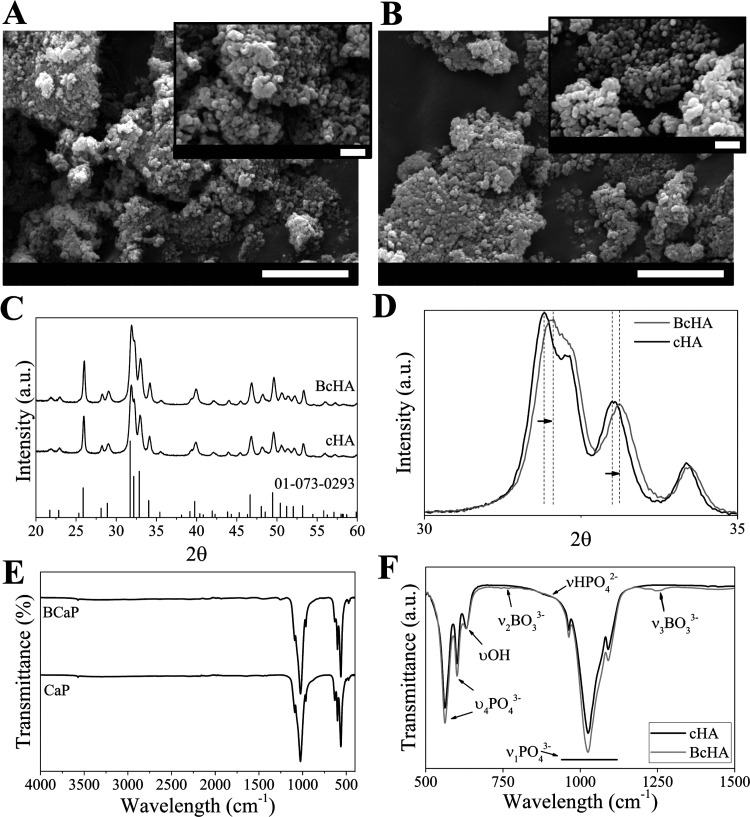
SEM images of cHA (A), BcHA (B) with close-up images in insets, their XRD spectra (C) and expanded spectra at 2θ between 30° and 35°, and FTIR spectra (E) and closer view of FTIR spectra between 500 and 1500 cm^–1^ (F). Inclusion of B in cHA lattice led to a shift in major cHA peaks at 31.9° and 32.98° to 32.06° and 33.08°, respectively. Scale bars are 2 μm.

BcHA was produced by the microwave reflux method. SEM analysis revealed that fine cHA and BcHA particles were obtained (Figure 2A,B). All samples showed spherical morphology; hence no difference between two samples was observed. Diffraction spectra demonstrated the formation of pure-phase hydroxyapatite in both cHA and BcHA (Figure 1C). In detail, a similar value of *X*_c_ (ca. 70%) was achieved (*p* = 1), and although no statistical difference was present, a decrease in *L*_c_ after B doping was observed (*p* = 0.088, Table 1). Significant microstructural differences were detected in the length of *a* = *b* axes, average V and D of the particle lattice (Table 1). Because the structure obtained after cHA and BcHA production was determined to be a hexagonal *P*63/*m* when compared to reference (PDF 01-073-0293), eqs 2, 3, and 4 were employed to determine lattice properties. It was determined that the B dopant could significantly decrease the *a* = *b* length (*p* < 0.001); thus *V* (*p* < 0.001) and *D* (*p* = 0.038) were smaller compared to cHA in return. In addition, a slight change in reflection plane 2θ values was observed, namely, the shifts from 31.9° to 32.06° and from 32.98° to 33.08° in BcHA compared to cHA in the specific peak range for HA lattice (Figure 2D). Also, the size of the crystals reflected at (0 0 2) and (2 1 1) decreased from 3.445 and 2.8187 Å to 3.4301 and 2.80 Å. Moreover, Fourier transform infrared (FTIR) spectra revealed that cHA and BcHA had hydroxyapatite-related functional groups such as 562 cm^–1^ ν_4_PO_4_^3–^, 603 cm^–1^ νOH, and a broad ν_1_PO_4_^3–^ band having a peak at 1027 cm^–1^ (Figure 2E). However, BcHA additionally showed the presence of B due to ν_2_BO_3_ and ν_3_BO_3_ at 760 and 1254 cm^–1^, respectively (Figure 2F). Because most of the HPO_4_^2–^ related peaks overlap with strong ν_1_ and ν_4_ vibrations of PO_4_^3–^ (around 550 cm^–1^ and 900 to 1100 cm^–1^), the presence of HPO_4_^2–^ was detected only by the small shoulder at 885 cm^–1^.^35^ It is also worth noting that the decrease in the sharpness and intensity of PO_4_^3–^ peaks and drastic change in νOH intensity were observed (Figure 2F).

**Table 1:** Microstructural Properties of cHA and BcHA (*n* = 3)

sample	*X*_c_	*L*_c_	*a* = *b*	*C*	*V*	*D*
cHA	69.87 ± 0.33	16.93 ± 1.79	9.84 ± 0.02^a^	6.86 ± 0.02	575.76 ± 3.86^a^	2.90 ± 0.02^a^
BcHA	69.46 ± 1.40	14.23 ± 1.07	9.74 ± 0.01	6.86 ± 0.02	563.13 ± 1.97	2.86 ± 0.02

a Statistically higher data obtained for cHA compared to that of BcHA at *p* < 0.001 for the length of *a* = *b* axes, *p* < 0.001 for *V*, and at *p* = 0.038 for *D*.

Further structural and elemental analysis demonstrated that cHA and BcHA particles had a similar agglomerate size (*p* = 0.825), Ca (*p* = 0.322), P (*p* = 0.219), and Ca/(P + B) content (*p* = 0.319). The statistical difference between samples was determined for the SSA (Table 2). BcHA displayed a significantly higher SSA compared to that of cHA (*p* = 0.28).

**Table 2:** Result of Particle Size (*n* = 3), ICP (*n* = 3), and BET (*n* = 3) Analyses

sample	*D*[4,3]	Ca (molar%)	P (molar%)	B (molar%)	Ca/(P + B)	SSA (m^2^/g)
cHA	12.01 ± 0.08	61.31 ± 0.28	38.69 ± 0.28		1.58 ± 0.02	58.31 ± 3.31
BcHA	12.27 ± 1.47	60.30 ± 1.52	37.20 ± 1.75	2.49 ± 0.23	1.52 ± 0.09	66.41 ± 2.51^a^

a BcHA resulted in a significantly higher SSA in the BET study (*p* = 0.028). No other significant differences were observed.

**Figure 3:**
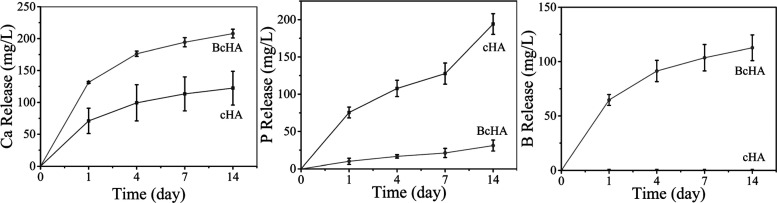
Cumulative release of Ca, P, and B from cHA and BcHA over time (*n* = 3). Ca release and B release from BcHA are significantly higher than those from cHA (*p* < 0.001), and P release from cHA is significantly higher than that from BcHA (*p* < 0.001).

Heat-sterilized powders of cHA and BcHA were weighed and placed in release medium as explained previously for the release study (2 g/10 mL). A statistically higher release of Ca was observed for BcHA as early as 1 day (*p* < 0.001), and this trend continued until the end of the analysis (Figure 3). P release from powders was the highest from cHA while almost 6 to 1 ratio was detected for P released from BcHA (*p* < 0.001). A time-dependent increase in B release from BcHA was also observed. BcHA induced significantly higher B release (*p* < 0.001) compared to cHA.

**Figure 4:**
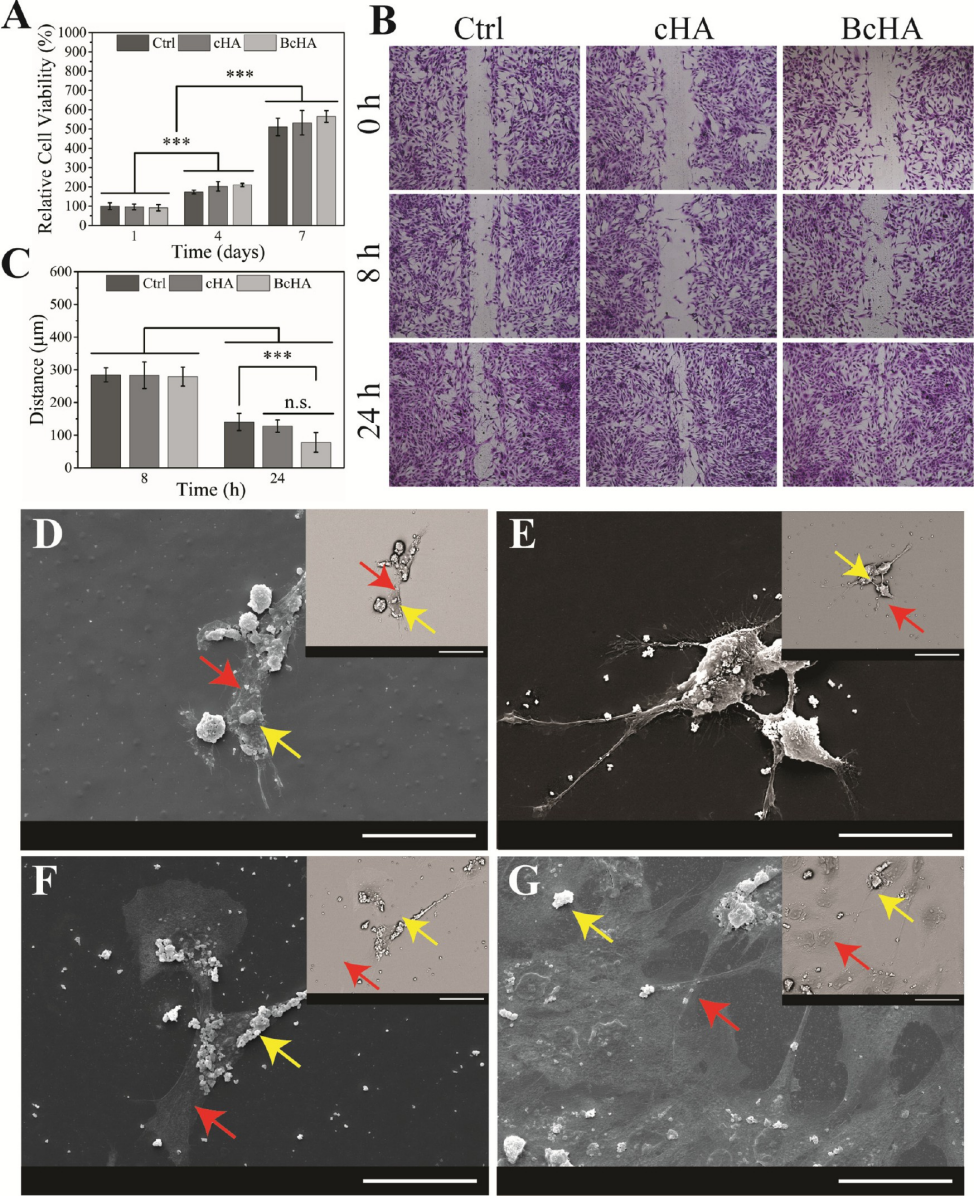
Relative hFOB viability (*n* = 8, A) at the end of 1st, 4th, and 7th days of incubation, migration of hFOB visualized by CV staining (B), and the change in the scratch distance with hFOB migration over time (*n* = 3, C) while cells were incubated with cHA and BcHA conditioned growth media for 8 and 24 h. SEM images of hFOB cells incubated in direct contact with cHA added to the media (100 μg/mL) and BcHA after 1 day (D and E, respectively) and 7 days (F and G, respectively) of incubation. Red arrows show cell body, and yellow arrows show cHA or BcHA particles. Scale bars are 50 μm. Insets in D–G are backscatter images which were obtained to increase contrast in between the cell body and powders.

Similar hFOB viability was observed for all groups at all time points (*p* > 0.05, Figure 4A). Moreover, cellular viability significantly increased with time for all samples (*p* < 0.001, each time point). In addition, hFOBs seeded on TCPS were scratched and analyzed for the change in the coverage scratch distance over time (Figure 4B). BcHA displayed greatest influence on cellular migration (*p* < 0.001 in comparison to Ctrl) within 24 h, despite no statistical difference compared to cHA (*p* = 0.07, Figure 4C). In addition to indirect analysis using cHA and BcHA conditioned media, these samples were also added directly on hFOBs (Figure 4D–G). The presence of cHA and BcHA both in as well as around the cells was detected. Cells were observed to interact with cHA and BcHA particles without any sign of structural damage; on the contrary, BcHA-treated hFOBs showed a number of cell to cell interactions (Figure 4E) and extensive lamellopodia growth at the end of the 7th day (Figure 4G).

**Figure 5:**
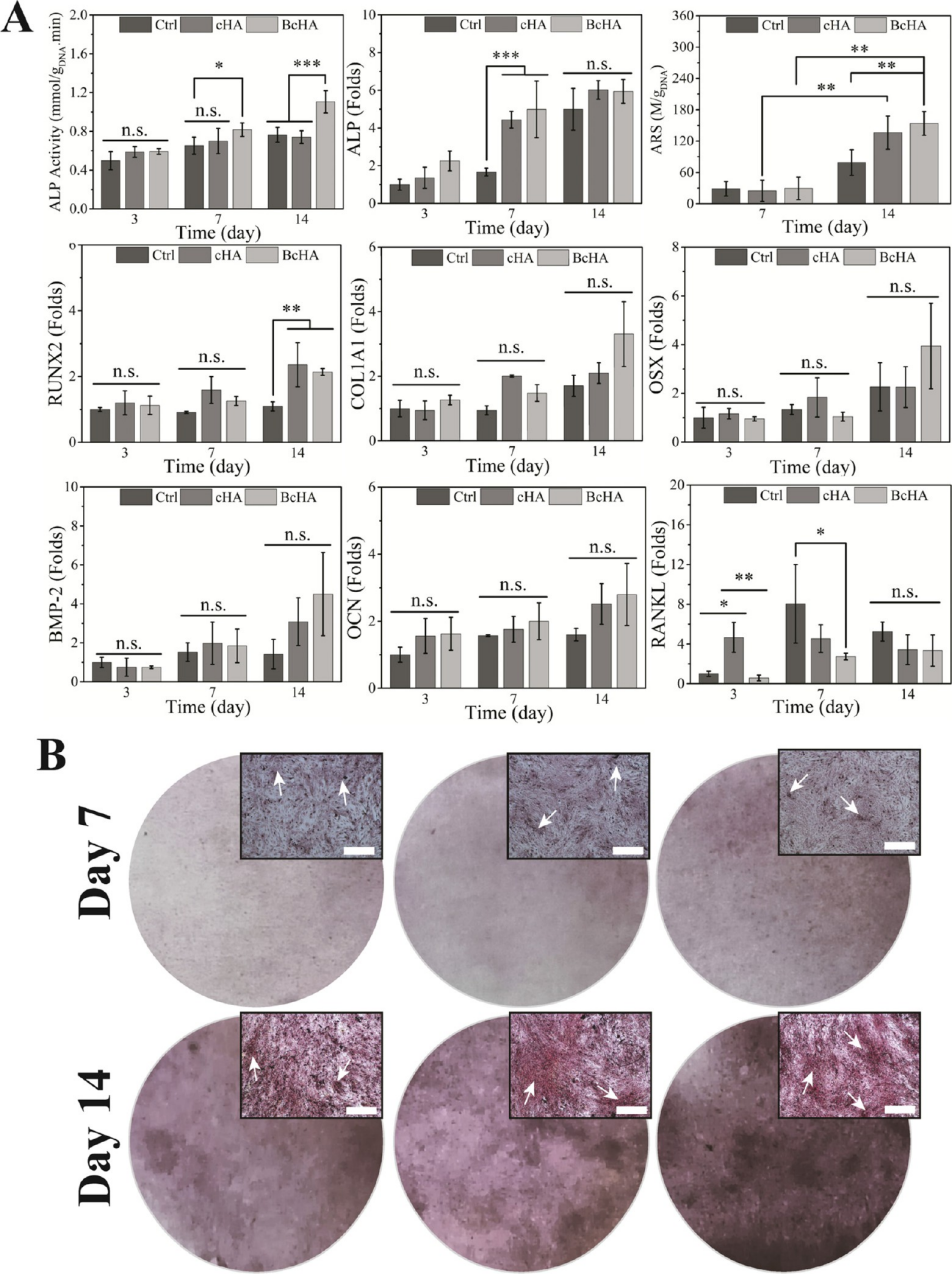
Results of hFOB ALP activity (*n* = 6), ARS content (*n* = 3), and qPCR analysis for osteogenic genes (*n* = 4) (A), and phase contrast microscope images obtained during the ARS study (B) after treatments with Ctrl, and extracts of cHA and BcHA. Statistical differences are denoted as *p < 0.05, **p < 0.01, ***p < 0.001, and “*n.s.*” for nonsignificant differences. Circular ARS images are conventional photos from six-well plates and rectangular images are phase contrast microscopy images obtained at 10× magnification. Scale bars of insets are 100 μm. White arrows in insets show CaP deposits stained by ARS.

After the cytotoxicity test of cHA and BcHA, osteogenic differentiation analysis was conducted (Figure 5A). At all periods, BcHA resulted in the highest ALP activity. This demonstrated statistically higher ALP activity than Ctrl (*p* < 0.05) and similar result to cHA at the end of day 7 and it also showed highest ALP activity (*p* < 0.001) compared to other groups at the end of the analysis (1.11 ± 0.11, *p* < 0.001). A gene expression study was conducted to ascertain the positive osteogenic effect of BcHA on hFOBs (Figure 5A). ALP expression was the highest on the third day of incubation and leveled off at the end of 14th day, with no statistical difference. At day 14, RUNX2 expression in cHA- and BcHA-treated hFOBs was similar which was significantly higher than the control (*p* < 0.01). Expressions of important late-stage marker genes such as COL1A1, OSX, BMP-2, and OCN were higher for BcHA-treated hFOBs compared to Ctrl and cHA; however, no statistical differences were obtained. RANKL expression was observed to be the highest in cHA-treated samples at the end of the third day (*p* < 0.001). However, Ctrl was the highest at the end of the seventh day (statistically higher than BcHA at *p* = 0.05) and 14th day, although no statistical differences were obtained. ARS was also conducted to determine the effect of BcHA qualitatively on biomineralization (Figure 5B). As early as 1 week, BcHA demonstrated a higher deposition of CaP when treated with BcHA extracts. The increase in darkness of color over time displays the increment in ARS intensity. White arrows in the image insets demonstrate CaP deposits. Moreover, in parallel with the change in color intensity over time, quantitation of ARS resulted in a steady increase of ARS from day 7 to day 14 (Figure 5A). Although all samples showed an increase in ARS intensity, only cHA and BcHA resulted in a significant change in CaP deposition (*p* = 0.008 and *p* = 0.002, respectively). In addition, it is important to note that BcHA-treated hFOB led to a statistically significant increase in CaP deposition compared to Ctrl (*p* = 0.008). This increment was higher than that of cHA treatment.

**Figure 6:**
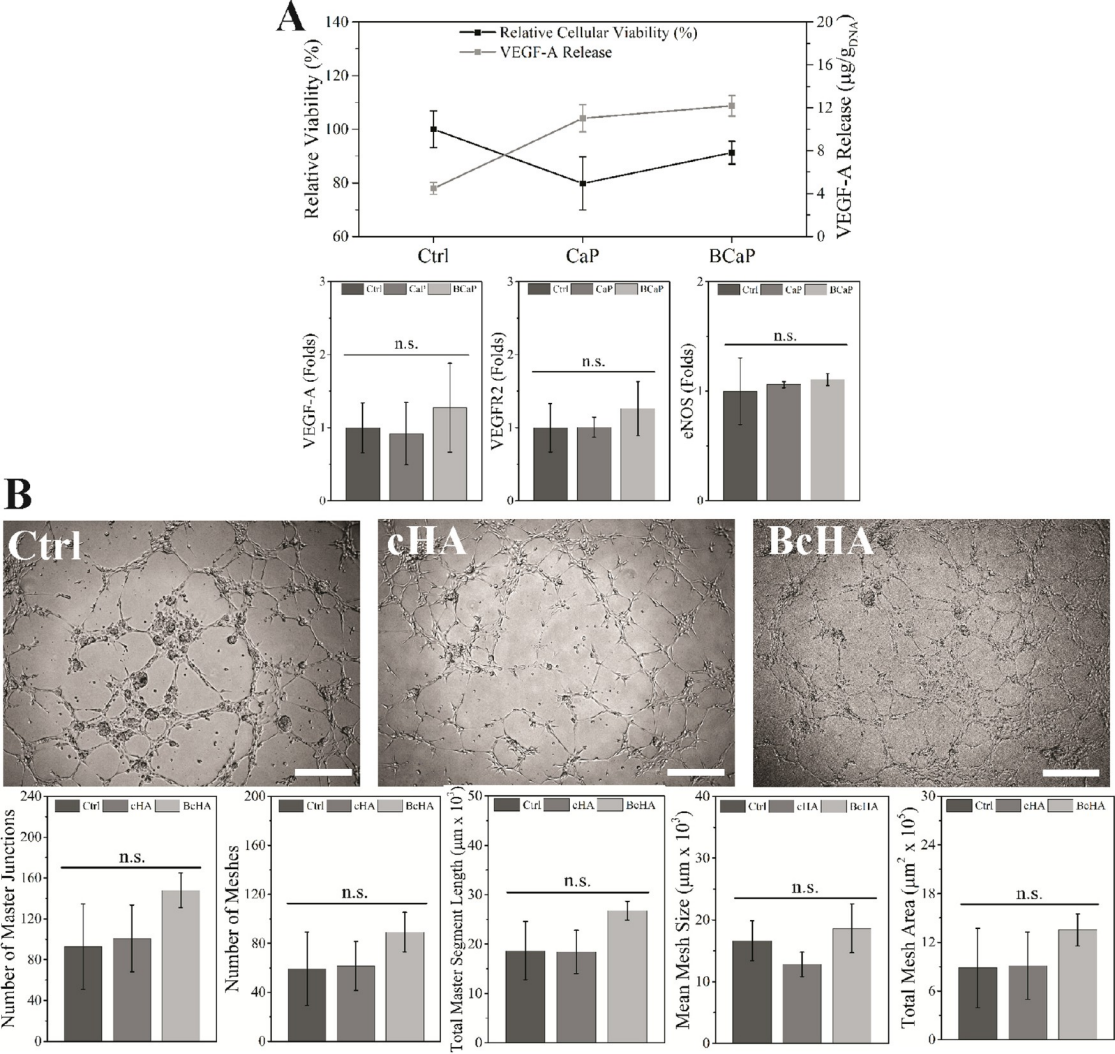
Amount of VEGF-A released by HUVEC (*n* = 4) and their cellular viability (*n* = 8), relative expression of angiogenic marker genes (*n* = 4, A), and tube formation analysis results (number of junctions, meshes, mesh sizes, segment lengths, and mesh areas, (B) after 4 h treatment with conditioned media (*n* = 6). Ctrl showed the highest cell viability (*p* < 0.001) and lowest VEGF-A release (*p* < 0.001). BcHA resulted in higher viability than cHA (*p* = 0.005), and similar VEGF-A release (*p* = 0.095) compared to cHA. BcHA also prompted the highest expression of angiogenic genes; however, it did not result in any significant differences. There was no statistical difference observed in tube formation analysis results despite all results being numerically higher for BcHA-treated HUVECs in all categories. Statistical differences are denoted as *p < 0.05, **p < 0.01, ***p < 0.001, and “*n.s.*” for nonsignificant differences. Scale bars are 100 μm.

Ctrl showed the highest HUVEC viability (*p* < 0.05) while BcHA and cHA showed similar cell viability results (*p* > 0.05) (Figure 6). On the other hand, VEGF-A release was observed to be immensely higher in cHA- and BcHA-treated samples compared to Ctrl (*p* < 0.001). Among cHA and BcHA samples, BcHA was found to trigger significantly higher VEGF-A release (1111.14 ± 87.82 in 4 h, *p* = 0.009) among the groups. Gene expression analysis revealed that BcHA treatment allowed HUVECs to express angiogenic genes in higher folds compared to cHA treatment and Ctrl (Figure 6). Similar to tube formation analysis results, there was no significant difference between groups.

HUVECs treated with cHA and BcHA and also in the Ctrl group showed the ability to form endothelial tubes (Figure 6). In particular, BcHA-treated samples displayed highly branched tube morphology. These cells were also able to form a greater number of endothelial meshes and segments than other groups. As a result, BcHA demonstrated highest results across all categories related to tube formation analyses (Figure 6).

**Figure 7:**
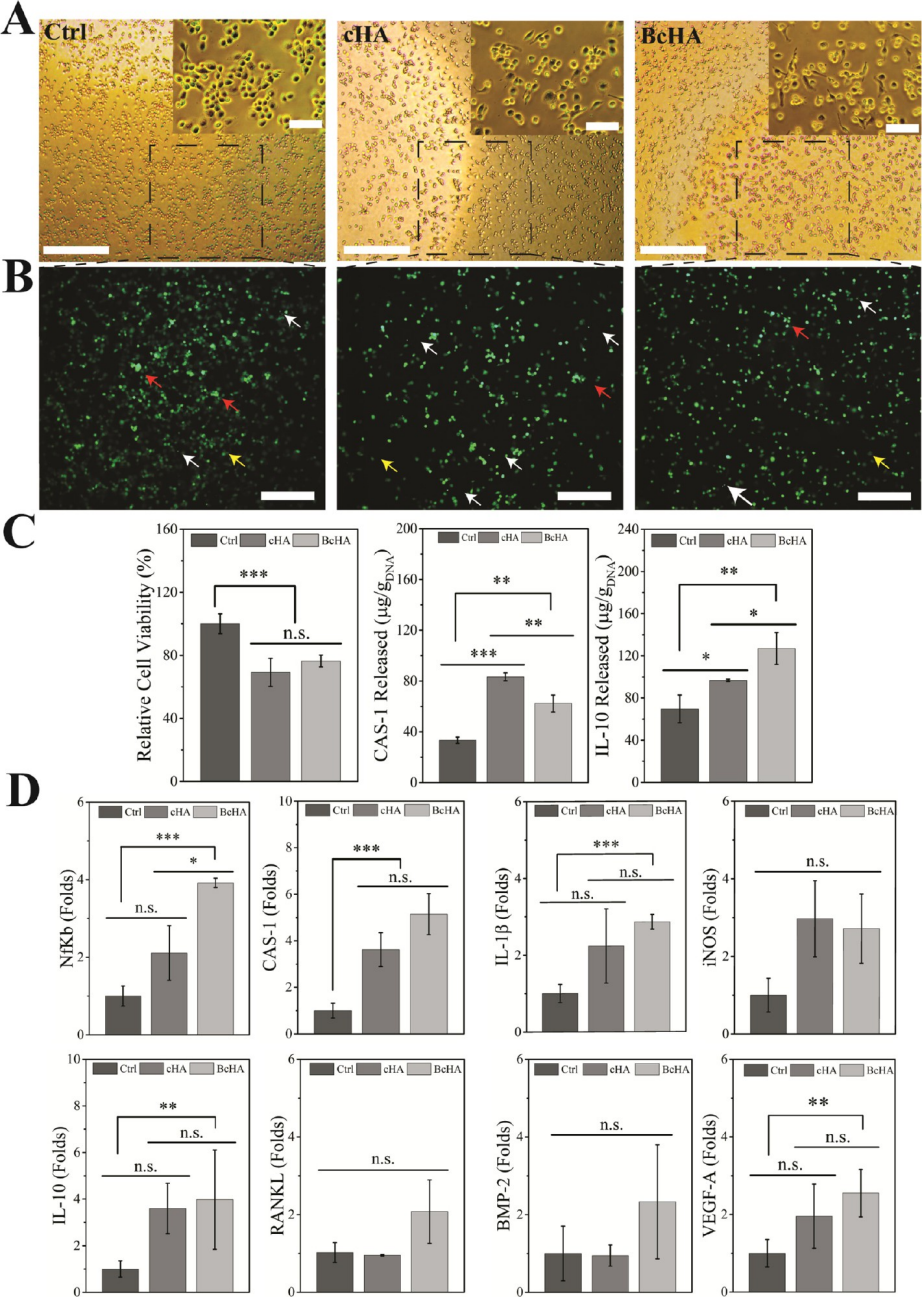
Phase contrast microscope images (A), fluorescence microscope images (B), and results of relative cell viability (*n* = 4), CAS-1, and IL-10 protein release (C, *n* = 3), and qPCR (D, *n* = 3) studies after Ctrl, cHA, and BcHA treatment of THP-1s. Statistical differences are denoted as *p < 0.05, **p < 0.01, ***p < 0.001, and “*n.s.*” for nonsignificant differences. Red arrows show stably expressed ASC without polymerization while yellow arrows demonstrate ASC agglomerates upon spontaneous activation (Ctrl) or after primed owing to the presence of ions released by cHA and BcHA. Scale bars in (A) are 500 μm in larger images and 50 μm in insets, and 300 μm in (B).

Phase contrast and CLSM images of Ctrl and cHA- and BcHA-treated THP-1s are given in Figure 7. Only PMA-treated samples appeared in round morphology while cHA and BcHA-treated cells had a spindle-like shape. THP-1s in Ctrl also demonstrated dilute ASC-conjugated structures without forming into small and bright ASC specks. Other samples, on the other hand, displayed a higher number of ASC specks formed after cHA and BcHA treatments.

Good THP-1 viability after incubation in the conditioned media was observed (Figure 7). Although viability of THP-1 significantly decreased (*p* < 0.001), no drastic cytotoxicity (like viability being <70% of Ctrl) was observed. Media collected after the incubation period were used to determine the concentrations of pyroptotic CAS-1 and anti-inflammatory IL-10 protein in the supernatant (Figure 7). Highest CAS-1 released was observed from THP-1s treated with cHA extract (95.98 ± 6.94 μg/g_DNA_) compared to Ctrl (*p* < 0.001) and BcHA extract (*p* = 0.008). In addition, BcHA extracts led to higher CAS-1 release (50.18 ± 5.52 μg/g_DNA_) compared to Ctrl (28.76 ± 4.59 μg/g_DNA_) at *p* = 0.002. In addition, BcHA caused highest IL-10 release (126.97 ± 15.05 μg/g_DNA_) compared to Ctrl (69.66 ± 13.17 μg/g_DNA_) at *p* = 0.009 and cHA (96.78 ± 1.07 μg/g_DNA_) at *p* = 0.026.

Expression of immunomodulatory genes and RANKL, BMP-2, and VEGF-A regenerative genes was analyzed (Figure 7). Inflammatory and pyroptotic NfKB expressions were found to be significantly the highest in BcHA-treated samples (*p* < 0.001 than Ctrl and *p* < 0.05 than cHA). No significant difference among Ctrl and cHA was detected. CAS-1 expression was not significantly different between cHA and BcHA, but they showed higher expression compared to Ctrl (*p* < 0.001). Expression of IL-1β, one of the proinflammatory genes, was the highest in BcHA-treated samples; however, no significant difference was observed between cHA and BcHA (*p* = 0.724). Similarly, cHA and Ctrl showed close results (*p* = 0.106) while BcHA triggered higher IL-1β expression than Ctrl (*p* < 0.001). Another proinflammatory gene, iNOS, expression was numerically higher in both cHA and BcHA compared to Ctrl. The anti-inflammatory IL-10 gene expression level was similar to that of IL-1β expression. BcHA led to significantly higher IL-10 gene expression than Ctrl (*p* < 0.01) while no statistical difference was obtained between cHA and BcHA (*p* = 0.297).

BcHA triggered higher expression of RANKL and BMP-2 genes; however, no statistical difference was observed. In the case of VEGF-A, BcHA triggered higher gene expression than Ctrl (*p* < 0.01), but no significant difference was detected between either cHA and Ctrl or cHA and BcHA.

## Discussion

Accounting for speed, scalability, reproducibility, and cost effectiveness, HT and BHT samples were produced by the microwave reflux method.^29^ In this study, fine HA particles with nanosized grains and phase purity were obtained (Figure 2). Samples had similar *X*_c_ around 70% and *L*_c_ around 15 nm; therefore they could be referred as moderately crystalline nanohydroxyapatites. With B doping, there was a significant decrease in *a* = *b* axes and no change in c axis dimensions inferring that B could be substituted along the c axis with PO_4_^3–^ and OH^–^ while leading to removal of Ca^2+^ from positions in *a* = *b* most probably as a result of charge compensation.^36^ The FTIR study showed that the B dopant was in the form of BO_3_^3–^ replacing the PO_4_^3–^ and OH^–^ as a result of dramatic decrease in OH^–^ intensity at 603 cm^–1^ and overall decrease in spectrum sharpness due to lower PO_4_^3–^ content.^37^ Furthermore, the decrease in axes lengths brought about lower *V* and lower *D*. Lower density upon inclusion of B is critically important because various methods have been meticulously studied to attain lower density in bioceramics to increase implant acceptance and solubility as well as to overcome the stress shielding phenomenon which prevents favorable mechanotransduction at the bone–biomaterial interface.^38^ In this context, BcHA could perform much more efficiently in comparison to cHA.

Agglomerates of cHA and BcHA had spherical morphology, and their particle size was determined assuming that all agglomerates were in the form of a perfect sphere.^39^ Employing *D*[4,3], it was determined that both samples showed an identical size. In addition, despite the fact that a significant change was observed in *a* = *b* axes upon B uptake, cHA and BcHA showed quite similar Ca and P content in their structure (Table 2). Interestingly, Ca/P or Ca/(P + B) was not statistically different. Therefore, it can be concluded that B uptake was successful and its rate could be monitored easily.^40^

On the other hand, the smaller size of B in comparison with P might have led to a decrease in the lattice size which resulted in an evident change in the SSA (Table 2). This change was not reflected in *L*_c_; however, the overall SSA was increased, leading to extensive Ca and B release (Figure 3). Moreover, replacing P at the periphery of the cage could allow efficient absorption and faster release of B compared to P. As discussed by Kolmas et al., B species could have been absorbed at the outer lattice; therefore, B release was significantly higher in comparison to P.^41,42^ Charge compensation, as demonstrated by a decrease in *a* = *b*, also led to the formation of HPO_4_^2–^ as shown in FTIR analysis. In addition, carbonate peaks were not observed in XRD and FTIR studies. Therefore, it can be speculated that calcium-deficient BcHA might bring about a vacancy in Ca sites and loss of PO_4_^3–^ after doping with anionic ions.^43^ Furthermore, Zahn and Hochrein showed that formation of Ca vacancies in cHA could be compensated with 1:1 change in HPO_4_^2–^:OH^–^ and every Ca vacancy may create two PO_4_^3–^ dissociation due to charge compensation in addition to compensation of Ca loss with dissociation of OH.^44^ For these reasons, B doping in cHA could be summarized in the following chemical reactions, where *x* demonstrates the molar content of HPO_4_^2–^ and *y* shows the molar content of BO_3_^3–^ in the structure:
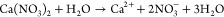
1
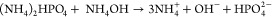
2
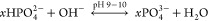
3
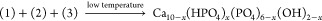
4
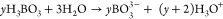
5

Finally, the overall chemical reaction can be given as follows:
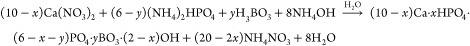


Proliferation and migration studies revealed that cHA and BcHA were biocompatible and did not show a significant change in the rate of proliferation over time. Nonetheless, these samples demonstrated an increase in cellular migration. hFOBs were observed to migrate at a faster rate when interacted with BcHA extracts than the other samples. In addition, direct contact analysis showed clear interaction of cells with the particles. Treatment of cells directly with BcHA allowed greater cell junctions to be formed and induced a higher number of cells to form lamellopodia. As reported elsewhere, cells may migrate and proliferate in interconnected clusters as a result of supportive mechanobiological influence of surface topography, bioactive ions, or coculture systems.^45,46^ Here, BcHA-treated hFOBs were found to exhibit similar spatial control over morphology of cells in terms of forming less spindle-like but more round and spread morphology compared to cells on cHA or Ctrl (Figure 4D–G). Hence, it can be said that the abovementioned findings corroborated the positive influence of BcHA on both direct and indirect contact with human osteoblasts.

Spreading freely with a greater cytoplasmic area tend to reinforce the osteoblastogenic differentiation rate as reported elsewhere.^47,48^ In addition, ALP expression was leveled off at the end of day 14 for cHA and BcHA. BcHA demonstrated a higher ALP gene expression although no significant difference was observed. Such an increase reached by BcHA could have been translated to higher ALP activity at the end of the incubation period. Additionally, induction of higher Ca^2+^ release as observed by the ICP study and prompting COL1A1 and OCN expressions by BcHA could have further enhanced ECM deposition of hFOB.^49,50^ Therefore, ARS analysis, both qualitatively and quantitatively, exhibited a higher intensity of CaP deposition around hFOB by BcHA.

The increase in RANKL expression in late stages allowing greater osteoblastic differentiation is the ideal case for achieving the most effective osseointegration.^51^ Thus, cHA may demonstrate a rapid osteoclast differentiation due to high RANKL expression while BcHA led to the lowest RANKL gene expression in 3 days. Higher RANKL expression related to early osteoclast differentiation was shown to be prompted by higher Ca^2+^ in ECM, and on the contrary, osteoclast differentiation could be suppressed by the presence of higher PO_4_^3–^.^52^ Moreover, the reverse relationship of RUNX2 and RANKL demonstrated a steady change in the RUNX2 decrease in comparison to Ctrl while RANKL increased in BcHA over time. Thus, it could be considered that BcHA might allow higher osteoblastogenesis and bone deposition which would be followed by the remodeling phase as RANKL expression would dominate RUNX2.^53^

In addition to RANKL expression, in spite of being statistically insignificant, higher ALP, COL1A1, BMP-2, OSX, and OCN gene expressions were achieved by BcHA. BcHA further demonstrated statistically the highest ALP activity and qualitatively better biomineralization (Figure 5). Together with the observations reported in Figures 4 and 5, increased spreading and a significant increase in ALP activity in addition to rise in transcription of osteogenic genes after being incubated with BcHA conditioned media further affirmed osteogenic potential of B when effectively suited as a bioceramic in cHA.

The angiogenic response of HUVECs after BcHA treatment demonstrated that the boron dopant could prompt tube formation and direct mesh formation as well as segmentation (Figure 6). A larger network volume, higher mesh segmentation, and a greater number of nodes were observed in the BcHA-treated group than Ctrl- and cHA-treated groups. This outcome also supported the data obtained for VEGF-A release.^54,55^ It is important to note that B and Ca release from BcHA resulted in a more efficient temporal modulation on VEGF-A as well as expression of angiogenic genes, such as eNOS and VEGFR2, compared to Ctrl or Ca amount P released from cHA. Therefore, it could be speculated that there should be a synergistic effect of B and Ca in rapid vascularization.^9,56,57^

A significantly lower THP-1 viability was detected without no cytotoxicity after treatment with extracts of cHA (from 100% to 69.21 ± 8.93%) and BcHA (from 100% to 76.47 ± 3.72%). Therefore, high viability obtained for both samples allowed us to interpret immunomodulatory protein release and qPCR data correctly, without underestimating the direct treatment of boron in BcHA.^58^ In addition, THP-1s were morphologically in both M1 and M2 state as observed in the phase contrast images. M2-specific spindle-like morphology was observed in all groups and none of the groups resulted in an enlarged and high number of filopodia presenting apoptotic macrophages. In addition, BcHA demonstrated a slightly higher number of M2-like morphology in comparison to rounded M1-like cells. As reported by Bai et al., macrophage morphology can be used to “qualitatively” estimate the overall reaction of macrophages in an osteoimmunomodulator environment.^59^ In addition, they concluded that highly osteoimmunomodulator conditions could lead to the formation of a balance between M1/M2 type; however, this balance might not be reflected by a morphological transition. Here, it can be concluded that the decrease in cell viability could be as a result of mounting the innate immune response leading to pyroptosis/efferocytosis in the presence of extracts and morphological transition could be a reporter of induction of macrophages toward the prohealing state.^60,61^

ASC is an adapter protein which forms a multiprotein assembly called speck and cleaves procaspase-1 into caspase-1 (CAS-1).^62^ Upon uptake of danger signals in innate immune cells, nuclear factor kappa B (NFκB), a regulator of innate immune response, is activated.^63^ NFκB triggers expression of proinflammatory cytokines such as CAS-1, iNOS, and IL-1β. Inflammasome multiprotein complexes are formed by NLRP3, ASC, and CAS-1 assembly.^64^ Inflammasome further elevates the inflammatory response as a result of cleaving pro-IL-1β into mature IL-1β.^65^ In addition to coincidental IL-1β production and CAS-1 activation, CAS-1 could induce pyroptotic cell death unless the inflammatory response is resolved. Therefore, determination of CAS-1 enzymatic activity by an ELISA study using a suitable substrate and ASC specks via visualization of GFP reporter could indicate inflammatory potential of BcHA. In the case of biomaterial application, the persistent inflammatory response plays a crucial role in the development of chronic inflammation which could lead to foreign body giant cell formation.^66^ Conversely, timed resolution of inflammation could prompt the prohealing cascade and hence improve the rate of implant integration as well as attaining functional bridging. Obtaining significantly lower THP-1 number demonstrates the active response brought about by BcHA treatment, probably because of efferocytosis/apoptosis.^67,68^ Further analysis revealed that BcHA resulted in significantly lower CAS-1 production than cHA, but it was detected to be higher than Ctrl. In addition, cHA and BcHA resulted in higher ASC speck presence showing greater stimulation compared to Ctrl.^69^ Concurrently, anti-inflammatory IL-10 production was the highest in BcHA-treated THP-1s. Therefore, this stimulation brought about by BcHA treatment demonstrates the effectiveness of BcHA in governing immunomodulation. In other words, it could be expected by BcHA application that the inflammatory response around the defect site could be quickly alleviated.

One of the key points observed in our study is that canonical NfκB pathway genes were upregulated after cHA and BcHA treatments, but this did not lead to a decrease in prohealing IL-10 expression and its translation (Figure 7). The evident intertwined relationships between the anti- and pro-inflammatory factors, such as expressions of iNOS, IL-1β, and IL-10 genes, were similar for cHA and BcHA and higher than Ctrl in comparison (Figure 7). As a proinflammatory enzyme, iNOS has been known to take part in tissue repair,^70^ and prohealing IL-10 improves mitochondrial activity and hence the M2 survival.^71^ In addition, IL-1β plays a role in aggravating the inflammatory response, yet, it was found to further improve the rate of mineralization.^72^ In parallel, increased IL-1β, which is a downstream effector in the NfκB signaling pathway, was determined to coincide with increased apoptosis and expression of greater folds of regenerative genes in our study. These findings were similar to the previous studies in the literature which involves in the direct positive effect of inflammatory cytokines. An increase in Ca release and also synergistic effect provided by B release are thought to be responsible for mounting an acute innate immune response which could be resolved in time and lead to a prohealing response in support of osseointegration.^73,74^

Another interesting finding in our study was the fact that regenerative genes such as RANKL, BMP-2, and VEGF-A were upregulated more upon BcHA treatment than Ctrl- or cHA-treated THP-1s (Figure 7). Taken together with the acute inflammatory response incited by BcHA, increased RANKL, BMP-2, and VEGF-A transcription manifested a multifunctional response brought about by THP-1s treated with BcHA extracts. During biomineralization, the increased CaP deposition rate prompts osteoclastogenesis encouraged with RANKL secretion by both osteoblasts and MΦs.^75^ It was further reported in the literature that ASC specks take part in tissue remodel and allow biodegradation of HA to fuse implant and bone.^76^ Moreover, it was demonstrated by various studies that the angiogenic response could allow rapid sprouting of present vessels into the defect site while increasing the rate of homing of precursor cells, it can be inferred that VEGF-A expression by MΦ could improve the integration at the implant/bone interface.^77^

These interesting findings further strengthen the notion that the acute response achieved with BcHA treatment, as evidenced by proinflammatory gene expressions, may result in a more balanced heterogeneous M1/M2 population.^78^ Here, the presence of M1 and M2 at the same time in the presence of BcHA was evident, and clearly, it was highly immunomodulatory in comparison to Ctrl and cHA. Modulation of M1/M2 markers and having a heterogeneous pro- and anti-inflammatory MΦs could allow mounting an acute but balanced osteoimmunomodulatory response by BcHA.^79^ M1s triggered by BcHA could resolve possible bacterial invasion through acute proinflammatory activity and favorable M2 transition may help alleviating the innate immune response in time to prompt the prohealing response.^80,81^ This transition could be facilitated with the BcHA-related increase in VEGF-A^82^ and BMP-2^83^ upregulation.

**Figure 8:**
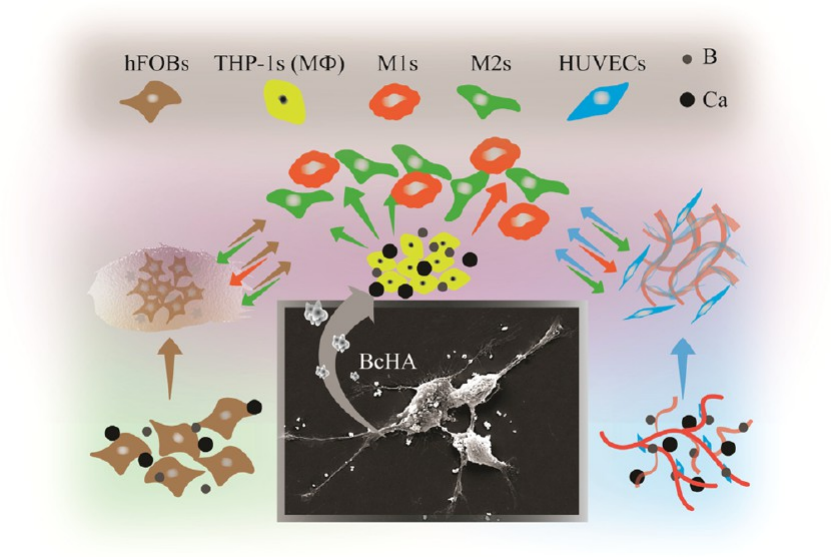
Cartoon shows the projection of the outcomes obtained in this study. B and Ca release provided from BcHA led to a M1/M2 heterogeneous population to form. These macrophages were determined to express regenerative genes; therefore, BcHA is expected to reinforce regenerative responses in addition to immunomodulation. BcHA also allowed a higher rate of osteogenic and angiogenic differentiation. Higher biomineralization around the hFOBs and rapid tube formation by HUVECs were also observed qualitatively after treatment.

Because B is a trace element, burst release in the vicinity of human cells could cause necrosis.^84^ Here, we introduced B into a cHA structure to allow sustained and more controlled release over time. Moreover, this structure was designed to be stable, less dense than its counterparts in the literature, easily produced, and scalable. Having B and Ca released concurrently also granted us the ability to study synergistic effect of Ca and B at the same time as multifunctional ions (Figure 8). It is critically important to note that higher Ca release achieved by BcHA besides B release could also be responsible for establishing a delicate balance between M1/M2 and also induction of regenerative answer.^85,86^ In this study, B in cHA demonstrated osteogenic, angiogenic, and immunomodulatory effects to support and encourage osseointegration.

## Conclusions

In this study, bioceramics were produced by microwave reflux in the form of cHA, and their osteogenic, angiogenic, and immunomodulatory potential has been investigated. BcHA was determined to has lower *L*_c_, *V*, and *D* compared to cHA. As a result, BcHA demonstrated a higher SSA and concurrent cumulative Ca and B release. Additionally, BcHA displayed better biomineralization and resulted in greater ALP activity and osteogenic gene expression. Release of B in addition to Ca from BcHA was shown to induce higher angiogenic activity in terms of promoting better tube network formation as well as higher VEGF-A release and angiogenic gene expression. Immunomodulatory studies revealed the potential of BcHA as a powerful osteoimmunomodulatory agent. BcHA was shown to incite an acute immune response which is strong and quickly transiting into the prohealing state by allowing higher release of CAS-1 and IL-10 proteins and expression of both pro- and anti-inflammatory genes. Real-time change in expression of various regenerative genes such as VEGF-A, RANKL, and BMP-2 upon BcHA incubation revealed an intricate osteoimmunomodulatory relationship in our study, parallel to the growing body of similar studies in the literature. Furthermore, it could be pointed out that the presented study successfully showed the merits of BcHA as a versatile and multifunctional bioceramic to steer the inflammatory response toward proinflammatory and then prohealing phenotype while encouraging osteogenesis and angiogenesis. It is also important to note that our work was limited to in vitro studies where we showed multifunctional properties of BcHA. Thus, an in vivo study BcHA will provide more insights about both local and systemic effects.

## Supporting Information Available

Equations (S1–S4) used in microstructural characterization of cHA and BcHA, media and cell types used in the study, and primers used in gene expression analyses (PDF)

## Notes

The authors declare that the data supporting the findings of this study are available from the corresponding author upon a reasonable request.
