# Supplementary material for: Versatile-in-All-Trades: Multifunctional Boron-Doped Calcium-Deficient Hydroxyapatite Directs Immunomodulation and Regeneration
Source: ACS Biomater Sci Eng. 2022 Jun 16;8(7):3038–53. doi: 10.1021/acsbiomaterials.2c00242 (PMC9277590; doi:10.1021/acsbiomaterials.2c00242)
Supplement: Supplementary file 1 — ab2c00242_si_001.pdf [file ab2c00242_si_001.pdf]

Supporting Information

Versatile-In-All-Trades: Multifunctional Boron Doped Calcium Deficient Hydroxyapatite Directs Immunomodulation and Regeneration

Authors/Affiliations

Ahmet Engin Pazarçeviren[1], Sema Akbaba[2], Zafer Evis[1] and Ayşen Tezcaner[1,3,*]

[1]Department of Engineering Sciences, Middle East Technical University, Ankara, Turkey

[2]Department of Biotechnology, Middle East Technical University, Ankara, Turkey

[3]Center of Excellence in Biomaterials and Tissue Engineering, Ankara, Turkey

*Corresponding Author:

Prof. Dr. Ayşen Tezcaner

Department of Engineering Sciences,

Middle East Technical University, Ankara 06800, Turkey

Tel: (+90) 532 494 56 91

e-mail: tezcaner@metu.edu.tr

Microstructural Characterization

Equations employed in calculation of microstructural properties of cHA and BcHA using XRD spectra:

$$X_C\ (\%) = \frac{Total\ crystalline\ peak\ area}{Total\ area\ under\ peaks} * \%100 \quad \text{(S1)}$$

$$\frac{1}{d(\text{Å})^2} = \left[\frac{4}{3} * h^2 + hk + k^2 + \frac{l^2}{(\frac{a}{c})^2}\right] * \frac{1}{a^2} \quad \text{(S2)}$$

$$Volume\ (V) = \frac{\sqrt{3}}{2} * a^2 * c \quad \text{(S3)}$$

$$Density\ (D) = \frac{W * Z}{V * 0.6022169} \quad \text{(S4)}$$

where d(Å) shows crystallite size (L_c) as obtained from the device, (h,k,l) shows Bragg indices, (a, c) shows axes in space. In density calculation, W shows molecular weight of given HA, Z shows number of molecular units in a given volume and 0.6022169 shows a constant derived from Avogadro's number.

Table S1. Media and cell types used in the studies.

Studies	Media	Cells
**Cell Proliferation, Migration, Direct Contact and, Osteogenesis Studies**	**Growth Medium:** DMEM:F12 (94.8%), fetal bovine serum (5%), 100U Penicillin-Streptomycin (0.2%).	Human fetal osteoblasts (hFOB)
	**Osteogenic Medium:** Growth medium supplemented with 10 mM β-glycerophosphate, 50 μg/mL L-ascorbic acid and 10^{-7} M dexamethasone.	
**Angiogenesis Studies**	**Angiogenic Medium:** EndoGo XF medium with supplements (Biological Industries, Israel).	Human umbilical cord vein endothelial stem cells (HUVEC)
**Immunomodulation Studies**	**Monocyte Medium:** RPMI 1640 (94.3%), FBS (5%), Normocin (0.2%), 100U Penicillin-Streptomycin (0.5%).<br>**Macrophage Medium:** RPMI 1640 (95%), FBS (5%) added with 50 ng/mL PMA**, without antibiotics.<br>**Conditioned Medium:** RPMI 1640 (95%), FBS (5%).	GFP conjugated ASC* carrying human monocytes (THP-1)

* ASC: Apoptosis-associated speck-like protein containing a pyrin domain and a caspase recruitment domain (CARD). In order to visualize, green fluorescent protein (GFP) fluorophore was conjugated.

** PMA: Phorbol 12-myristrate 13-acetate.

Table S2. Primers used in gene expression analyses. Primers were designed and tested using BLAST (NIH, USA) and Primer3 software.

Gene	Forward Primer (5' – 3')	Reverse Primer (5' – 3')	Accession Number
**ALP**	ATGGGATGGGTGTCTCCACA	CCACGAAGGGGAACTTGTC	NM_001127501.4
**OSX**	TGCTTGAGGAGGAAGTTCAC	AGGTCACTGCCCACAGAGTA	NM_152860.2
**OCN**	CGCTACCTGTATCAATGGCTGG	CTCCTGAAAGCCGATGTGGTCA	NM_199173.6
**RUNX2**	CCCAGTATGAGAGTAGGTGTCC	GGGTAAGACTGGTCATAGGACC	NM_001015051.4
**COL1A1**	GATTCCCTGGACCTAAAGGTGC	AGCCTCTCCATCTTTGCCAGCA	NM_000088
**BMP-2**	CTAAGGAGGACGACAGCACC	AAGAAGTCCCCAGCCAAGTG	NM_001200.4
**RANKL**	TGGAGCAATTACGGGGTGAC	GCGCTAGATGACACCCTCTC	NM_003701.4
**VEGF-A**	TCACCAAGGCCAGCACATAG	CCGGGATTTCTTGCGCTTTC	NM_001025366
**VEGFR2**	CTCCCTGCCGTGTTGAAGAG	TGAACCTCCCGCATTCAGTC	NM_002253
**eNOS**	GAAGGCGACAATCCTGTATGGC	TGTTCGAGGGACACCACGTCAT	NM_000603
**NfKB**	GCAGCACTACTTCTTGACCACC	TCTGCTCCTGAGCATTGACGTC	NM_001382627
**CAS-1**	GCTGAGGTTGACATCACAGGCA	TGCTGTCAGAGGTCTTGTGCTC	NM_001257119
**IL-1β**	CCACAGACCTTCCAGGAGAATG	GTGCAGTTCAGTGATCGTACAGG	NM_000576
**iNOS**	GCTCTACACCTCCAATGTGACC	CTGCCGAGATTTGAGCCTCATG	AF068236.1
**IL-10**	CATCAAGGCGCATGTGAACTC	AATCGATGACAGCGCCGTAG	NM_000572
**β-Actin**	CACCATTGGCAATGAGCGGTTC	AGGTCTTTGCGGATGTCCACGT	NM_001101
